# A Novel Approach for Multichannel Epileptic Seizure Classification Based on Internet of Things Framework Using Critical Spectral Verge Feature Derived from Flower Pollination Algorithm

**DOI:** 10.3390/s22239302

**Published:** 2022-11-29

**Authors:** Dhanalekshmi Prasad Yedurkar, Shilpa P. Metkar, Fadi Al-Turjman, Thompson Stephan, Manjur Kolhar, Chadi Altrjman

**Affiliations:** 1 Department of Computer Science and Engineering, MIT Art Design and Technology University, Pune 412201, India; 2 Department of Electronics and Telecommunication Engineering, College of Engineering Pune, Pune 411005, India; 3 Artificial Intelligence Engineering Department, AI and Robotics Institute, Near East University, Mersin 10, Turkey; 4 Research Center for AI and IoT, Faculty of Engineering, University of Kyrenia, Mersin 10, Turkey; 5 Department of Computer Science and Engineering, Faculty of Engineering and Technology, M. S. Ramaiah University of Applied Sciences, Bangalore 560054, India; 6 Department of Computer Science, College of Arts and Science, Prince Sattam Bin Abdulaziz University, Al-Kharj 16278, Saudi Arabia; 7 Faculty of Engineering, University of Waterloo, Waterloo, ON N2L 3G1, Canada

**Keywords:** channel, critical spectral verge, epilepsy, flower pollination, Internet of Things

## Abstract

A novel approach for multichannel epilepsy seizure classification which will help to automatically locate seizure activity present in the focal brain region was proposed. This paper suggested an Internet of Things (IoT) framework based on a smart phone by utilizing a novel feature termed multiresolution critical spectral verge (MCSV), based on frequency-derived information for epileptic seizure classification which was optimized using a flower pollination algorithm (FPA). A wireless sensor technology (WSN) was utilized to record the electroencephalography (EEG) signal of epileptic patients. Next, the EEG signal was pre-processed utilizing a multiresolution-based adaptive filtering (MRAF) method. Then, the maximal frequency point at which the power spectral density (PSD) of each EEG segment was greater than the average spectral power of the corresponding frequency band was computed. This point was further optimized to extract a point termed as critical spectral verge (CSV) to extract the exact high frequency oscillations representing the actual seizure activity present in the EEG signal. Next, a support vector machine (SVM) classifier was used for channel-wise classification of the seizure and non-seizure regions using CSV as a feature. This process of classification using the CSV feature extracted from the MRAF output is referred to as the MCSV approach. As a final step, cloud-based services were employed to analyze the EEG information from the subject’s smart phone. An exhaustive analysis was undertaken to assess the performance of the MCSV approach for two datasets. The presented approach showed an improved performance with a 93.83% average sensitivity, a 97.94% average specificity, a 97.38% average accuracy with the SVM classifier, and a 95.89% average detection rate as compared with other state-of-the-art studies such as deep learning. The methods presented in the literature were unable to precisely localize the origination of the seizure activity in the brain region and reported a low seizure detection rate. This work introduced an optimized CSV feature which was effectively used for multichannel seizure classification and localization of seizure origination. The proposed MCSV approach will help diagnose epileptic behavior from multichannel EEG signals which will be extremely useful for neuro-experts to analyze seizure details from different regions of the brain.

## 1. Introduction

Epilepsy is the most common neurological illness, impacting 50 million people globally [1]. Epilepsy is exhibited by recurrent seizures in the brain due to its association with the central nervous system. EEG signals are used to explore the functioning of the brain during an epileptic seizure [2]. The cause of an epileptic seizure is due to the presence of uncontrolled potential interruption in the brain. These abruptly occurring seizures are tricky and may lead to very dangerous situations [2]. Epileptic persons are three times more prone to die early as compared to a non-epileptic human being [1]. In addition, epileptic seizures generally start and end instinctively without any exterior intrusion. Also, they may remain unobserved. Hence, the analysis and detection of epileptic EEG signals has always been of great interest to researchers.

In view of this, continuous monitoring of real-time signals, such as EEG, is performed by employing WSN technology, one of the most promising emerging techniques for real-time monitoring of patients remotely. WSN utilizes body sensor network (BSN) that records vital signs of an epileptic patient such as EEG. A huge amount of data is generated with the rise in the use of BSN. Cloud computing (CC) facilitates storing and analyzing such rapidly produced EEG sensor data in real time from different patients located in distinct geographic regions. CC combined with BSN facilitates a framework to monitor and analyze the EEG data efficiently, in real time [3].

The main aim of this work was to develop an efficient cloud-based epileptic seizure monitoring system to help neuro experts automatically classify epileptic seizures. The proposed automated seizure monitoring system will assist neuro experts as an additional tool to take decisions with more precision. The objectives of this paper were to capture high frequency oscillations (HFO) related to seizure information for seizure classification using the CSV feature in real time; and employ the FPA technique for optimization of the CSV feature, thereby facilitating localization of the brain focal region where the seizure has occurred. In order to obtain these objectives, we have developed a model employing BSN, mobile phone, and cloud infrastructure.

The proposed work is presented as given below: Section 2 explains the literature; the technique utilized for the prediction of epileptic seizures is described in Section 3. An exploration of the results for multichannel classification of the epileptic seizure is presented in Section 4. Section 5 discusses the proposed approach; a comparative study of the proposed MCSV approach is detailed in Section 6 to predict seizures. Section 7 concludes the paper.

## 2. Literature

The literature work is divided into two sections. The first section discusses various feature extraction techniques used for the classification of epileptic seizures. The second section details the use of sensor technology by utilizing WSN and CC in the seizure classification.

### 2.1. Epileptic Seizure Classification Techniques

Various EEG analyses show that dynamic variations of brain activity occur in relation to time, frequency, and space parameters. In regard to a non-linear signal, frequency-related techniques are employed to analyze the working of the epileptic EEG data processing [4,5,6].

Research has been carried out on the diagnosis of seizures from long-term EEG recordings [7]. Time-varying methods and time-frequency methods have been proposed to interpret the EEG seizure frequency spectra during non-seizure and ictal periods. Various 2techniques studied for analyzing epileptic rhythms and spectral sub-bands include: dominant frequency representations [8], adaptive-based techniques [9], decomposition based on discrete wavelet transform (DWT) [10,11]; independent component analysis (ICA) [12], and multi-dimensional systems [13]. The interpreted features thus obtained are further analyzed by employing statistical methods.

Automated seizure detection methodology can be carried out either on a single channel data or on multichannel information [14,15]. In a single channel-based seizure detection mechanism, the correct selection of the EEG channel containing the strongest seizure information is very difficult. To obtain better seizure detection, seizure information contained in multichannel EEG signals is required to be processed effectively to extract spatial- temporal related knowledge [16]. Various studies have been proposed to find the correlation between multichannel data to spot the most prominent features and information network among different regions of the brain [17]. In [18,19], a weighted multichannel approach was utilized to extract the maximum power present in different frequency ranges. Other multichannel epileptic data processing methods were proposed in [20].

In order to interpret seizure information effectively and efficiently, a segment-wise analysis is proposed in literature. Kiranyaz et al. have used a segment-wise seizure detection strategy in lengthy EEG signals along with Particle Swarm Optimization (PSO) [21]. This approach has attained an average true positive rate of 89%, and an average false rate of 93%. In a similar line, Dong Wang et al. employed a single channel-segment wise seizure identification algorithm for lengthy EEG data [22]. This work has attained an average seizure detection rate of 95.82% [22].

A majority of the state-of-the-art studies that have been undertaken perform automatic diagnosis on single channel EEG signals. These techniques have overlooked correlations between the various channels which is clinically baseless [22]. Recently, deep learning networks have shown better performance in the classification of various EEG abnormalities. However, these techniques are unable to localize the seizure region [23]. Multichannel EEG recordings have a significant role in the recognition of seizure activities from the lobes of the brain. Automated computer-aided multichannel seizure diagnosis will assist neurologists in locating the specific area of the brain lobes where the seizure activity is taking place. Moreover, such automated diagnosis will prevent false alarms and result in the neurologists making educated and increasingly accurate decisions.

The work presented in this research paper has come up with a technique to detect epileptic seizures automatically from multichannel EEG recordings segment-wise and to localize the seizure region of the brain accurately. The proposed approach extracts the actual seizure information using the optimized MCSV feature by employing a bio-inspired (BI) algorithm, namely, FPA.

Clinically, visual interpretation of the epileptic EEG data is performed by brain experts. However, the task of examining these long-term seizure signals is very exhausting, time consuming, and difficult to diagnose. EEGs need to be analyzed by a fully automated computer-based system so that such limitations can be eliminated. It is also challenging to classify the exact epileptic seizure region in long duration EEG recordings. In this paper, multichannel segment-wise seizure classification was performed using FPA [24] which also helps to find the exact location of the occurrence of epileptic seizures.

### 2.2. Sensor Technology in the Seizure Classification

Currently, many research studies have explored the usefulness of integrating the WSN with the CC framework. However, very few research efforts have been undertaken to establish the feasibility of integrating cloud-enabled frameworks with BSN, facilitating online and offline monitoring of epileptic seizure events. Forkan et al. employed a model using a service-oriented framework to enable an assisted living service in real time. It employed a middleware layer that reduces the complexity of real time data from various distinct sensors and contextual information [25]. The authors of [26] have proposed an architecture using BSN and CC infrastructure. The aim of this research work was to monitor assisted living by adopting a wearable sensor that sends data to the cloud by using a mobile phone. Research in [27] has presented a prototype for collecting sensor information efficiently from wireless networks namely, the body area network (BAN). It employed a virtual machine and virtualized cloudlet. Recently, Lounis et al. employed a secure cloud-based framework based on WSN to process real time data for patients under critical supervision [28].

## 3. Methodology

**Figure 1:**
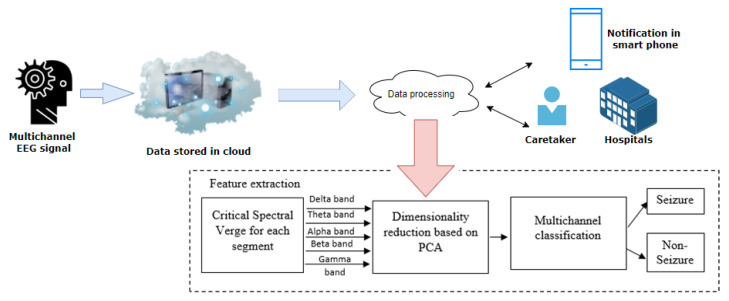
Block diagram of MCSV multichannel-based epileptic seizure classification.

The block schematic of the proposed multichannel seizure classification using the cloud storage and processing, and FPA approach is represented in Figure 1.

In the proposed approach, cloud storage and the processing part consist of distinct modules such as pre-processing and data management, feature extraction and dimensionality reduction, and multichannel classification. The IoT part comprises a smart phone and cloud to capture and transmit data blocks. The smart phone is connected through a proper communication protocol to the cloud server.

In the data processing module, initially the multichannel epileptic EEG data is pre-processed segment-wise using MRAF [29]. It is followed by the extraction of an optimized CSV feature using FPA and data dimensionality reduction using principal component analysis (PCA). Finally, the attained best feature set is transferred through to classifiers such as K-Means clustering, k-Nearest Neighbour (k-NN), and SVM for classification.

### 3.1. Segmentation of EEG Signal

The input EEG signal read from the multichannel is split into segments. For segmentation, a sliding window approach is used which breaks up the raw EEG data into sections or segments for feature extraction [24]. In the proposed work, the sliding time window for the EEG signal is 1040 data points (4 s) without any overlaps. The proposed MCSV approach is carried out for each EEG segment, for every frequency band such as delta, theta, alpha, beta, and gamma in the multichannel EEG signal.

### 3.2. MRAF-Based Pre-Processing

Frequently, EEG seizure details are affected by the presence of physiological artifacts [3]. So, it is very crucial to reduce such EEG interferences which hamper identification of the epileptogenic zone accurately. MRAF helps to remove the physiological artifacts by preserving the information of the epileptic seizure [29].

MRAF is implemented in three main steps:

(i) To begin with, the EEG signal that is input is decomposed into multiple frequency levels making use of DWT;
(ii) Next, soft thresholding is brought into use with the DWT coefficients to generate a signal with minimized abrupt changes;
(iii) Finally, MRAF is undertaken to produce an EEG signal that contains minimal physiological EEG artifacts.

Here, soft thresholding is executed on the obtained high frequency wavelet coefficients. Soft thresholding is rendered by (1) as follows [29]:

(1) $$T_{l}=\sigma_{l}\sqrt{2logR}$$

where $T_{l}$ is the threshold number, $\sigma_{l}$ is the standard deviation of the input signal, l is the limit of the DWT decomposition, and R is the number of samples of the EEG.

MRAF is then performed to produce the pre-processed information of the seizure zone. The data thus obtained is expressed by the MRAF expression [29]:

(2) $$X_{o}{\left(n\right)}_{per\;\left(b\right)}=\sum_{i=0}^{s-1}w_{i}\left(n\right)x_{i}\left(n-j\right)\;$$

where $X_{o}{\left(n\right)}_{per\;\left(b\right)}\;$ is the output of MRAF which can be obtained for each band b. Here, bands such as delta, theta, alpha, beta, and gamma are considered at the output stage, where all bands represent different resolutions of frequency and hence multiresolution filtering is achieved. $w_{i}\left(n\right)$ represents the weight of the MRAF approach and represents the newly adapted components of the adaptive filter.

### 3.3. Proposed Feature Extraction Technique

After pre-processing the EEG signal using MRAF, features are extracted for each EEG segment. The first step of feature extraction is the computation of the average of spectral values (average PSD) for each frequency band such as delta, theta, alpha, beta, and gamma. For each frequency band, for each segment, the PSD is calculated. Then the maximal frequency point at which the PSD of the segment is greater than the average PSD of the corresponding frequency band is computed which is termed as spectral verge (SV). However, due to the involvement of undesired glitches in the low frequency region most of the seizure related information is missed. In order to ensure that no seizure details are missed, the SV is optimized further using the FPA. Then, by considering the SV point as the seed point, optimization is performed by the FPA utilizing Levy’s path. The optimized point thus obtained is called the CSV. The CSV is obtained for each segment of the multichannel EEG signal.

Important steps for the computation of CSV given below:

Step 1: Process segment k of an EEG signal
Step 2: Process band b of an EEG signal
Step 3: Calculate power spectral values of all segments and all bands.
Step 4: Take the average of the calculated power spectral values.
Step 5: Apply the FPA to optimize and calculate the CSV.
Step 6: Add values of each calculated CSV to the feature map.

### 3.4. Mathematical Model

As per Equation (2), we have achieved multiresolution filtering. The filtered output $X_{o}{\left(n\right)}_{per\;\left(b\right)}$ can be named as *x (n)*, for simplicity. Then *x (n)* is split segment-wise for feature extraction. *x (n)*, is represented as ‘x’ where ‘n’ represents the sample index which is omitted. Now we represent the signal ‘x’ in the form of segments as:

Let $x=\left\{x_{1},x_{2},\ldots ,\;x_{k}\right\}$ where, k represents the segment index.

(3) $$Number\;of\;segments,\;k=\frac{Number\;of\;samples,\;n}{f_{s}\times segment\;size}$$

where $f_{s}$ is the sampling frequency.

The filtered signal in each band can now be spilt in a similar manner and the process is repeated for all frequency bands.

Now, the average spectral power is computed for each segment as uniform distribution of spectral power is observed for a high frequency range for the epileptic seizure segment.

Let $PS_{kb}$ be the power spectrum of the segment $x_{k}$, for band b where $PS_{k}$ can be expressed as $PS_{k}=\left\{PS_{kb}\left(0\right),\;PS_{kb}\left(1\right),\ldots ,PS_{kb}\left(m\right)\right\}$ and m = 0,1,…,$\;f_{s}$. For example, the power spectrum contains 250 points if f_s = 250 Hz then m = 0,1,…,$\;f_{s}$ are the frequency points between 0 to $f_{s}$. The power spectrum has power spectral densities of all frequency points between 0 to $f_{s}$ Hz. The average spectral power is expressed as:

(4) $$PS_{avg}=\sum_{i=0}^{m}\frac{PS_{kb}\;\left(i\right)}{f_{s}}$$

Now, we are interested in frequency point ‘m’ between 0 to $f_{s}$ Hz which satisfies the following conditions:

Condition 1: Power spectral density of that frequency point is greater than $PS_{avg}$.

Mathematically, if $m_{s}$ is the set of frequencies for which

(5) $$m_{s}=\{m\in PS_{kb}\left(m\right)>PS_{avg}\}\;$$

Condition 2: The frequency point ‘m’ itself is greater than any other point that satisfies condition 1.

Mathematically, if $max(m_{s})$ is the spectral verge point for which

(6) $$spectral\;verge=max(m_{s})$$

In the presented analysis, we have considered this spectral verge point as a criterion for classifying seizure activities. However, due to patient-specific frequency variations present in the seizure region, misleading frequency points in some segments lead to misclassification. In order to optimize this, we chose FPA as a swarm-based intelligence technique inspired by the pollination behavior of flowering plants. In order to solve this optimization problem, the primary requirement for FPA is initialization of the objective function f(x) and seed solution. We have considered a combination of (5) and (6) as our objective function which is as follows:

(7) $$max\left\{m\in PS_{kb}\left(m\right)>PS_{avg}\right\}$$

A seed solution was obtained from the objective function and used as input for the first iteration as the best fit solution.

We can state the solution function in a generalized form as $f_{si}$ to simplify the process further in terms of the FPA algorithm.

Now we need to optimize this seed solution to get the best fit using the pollination method as explained in the algorithm below:

FPA uses the concept of a biotic process of global and local pollination to search for the best fit, which we have used anonymously as global and local seizure abnormalities. In order to differentiate between the local and global seizure abnormalities, FPA iterates through local and global abnormalities effectively and optimizes the solution to correctly identify the epileptic seizure segments using the pollination algorithm.

The optimal solution is defined by:

(8) $$\;S_{i}=bestfit\;\left\{f_{si}\right\}$$

where i=1,2,…,d are the decision variables which are expected to be from a possible range of 0-fs.

So, the resultant solution will be in the upper frequency bound (fs) to the lower frequency bound (0 Hz).

The criterion for the best fit is the solution for which stability is achieved over possibilities of all solutions or maximum iterations are reached for solution generation.

In order to reach the best solution, define the population function map given as:

(9) $$Solution\;map=\left\{S_{i}\right\}$$

where $S_{i}$ is the set of solutions from the map to iterate.

FPA can now be applied to the population of solutions above to get the optimized solution.

A biotic or abiotic process of search for the optimized solution from the map of $S_{i}$ is performed by evaluating the relation with the current best fit solution:

The biotic process is given as:

(10) $$x_{i}{}^{\left(t+1\right)}=x_{i}^{\left(t\right)}+L\left(g^{*}-x_{i}^{\left(t\right)}\right)$$

where g* is the current best solution; $x_{i}\;$ represents the pollen or current solution vector; $x_{i}{}^{\left(t+1\right)}\;$ is the next solution; L refers to the strength of pollination derived using the Levy flight to mimic the distance step characteristic of each pollen.

Levy’s distribution is expressed as:

(11) L~λ γ(λ)sin(πλ2)π (S1+λ)

where *γ*(*λ*) is the gamma function with *λ* = 0.5.

The abiotic process is given as:

(12) $$x_{i}{}^{\left(t+1\right)}=x_{i}^{\left(t\right)}+\in \left(x_{i}^{\left(t\right)}-x_{k}\left(t\right)\right)$$

At each iteration the probability switch is calculated using random distribution to define which path to follow, local or global pollination.

There can be various stopping criteria for stopping the iteration. Here, we have chosen a finite number as the maximum iteration to achieve stability.

Finally, we get the best solution as the CSV which is the optimized solution of all the solutions in the map.

Now the CSV can be obtained similarly for all the EEG segments as $CSV_{1},\;CSV_{2},\ldots ,\;CSV_{n}$ for each segment.

For each band, alpha (*α*), beta (*β*), gamma (*γ*), theta (*θ*), delta (*δ*), and full band (*fb*), the CSV can be obtained in a similar fashion. The feature map thus formed can be represented by (13) as:

(13) $$\left[\begin{array}{c} CSV_{\alpha 1},CSV_{\alpha 2},\ldots ,\;CSV_{\alpha k}\\ CSV_{\beta 1},CSV_{\beta 2},\ldots ,\;CSV_{\beta k}\\ CSV_{\gamma 1},CSV_{\gamma 2},\ldots ,\;CSV_{\gamma k}\\ CSV_{\theta 1},CSV_{\theta 2},\ldots ,\;CSV_{\theta k}\\ CSV_{\delta 1},CSV_{\delta 2},\ldots ,\;CSV_{\delta k}\\ CSV_{fb1},CSV_{fb2},\ldots ,\;CSV_{fbk} \end{array}\right]$$

This CSV as an optimal feature map represents the signal relation between each EEG segment from 1 to k, each frequency band ranging from α to fb and EEG channels. This research proposes the computation of this CSV-based optimal feature map.

### 3.5. Feature Dimensionality Reduction

The features extracted by the proposed MCSV approach reflect the connectivity between each EEG segment, and EEG channels at different frequencies. Then, the MCSV values from channel 1 to channel N at frequency ‘f’ are acquired for each of the EEG segments. If we consider data from 21 channels, then, the 21 × 21 feature matrix for every frequency is extricated from the EEG signals received originally. When CSV values are dealt with directly, the outcome of the detection of seizure can be unsatisfactory as the complexity of the computation is very high making it very difficult to distinguish between the segments of the EEG signals that contain seizure information and those segments that do not [28]. Hence, it is very critical for a reduction in feature dimensionality to enhance the generalization capability of the classifier and make designing it easier. PCA establishes a new coordinate system for the EEG data, such that each data point can be identified by a combination of orthogonal components [24]. The dimensionality of the extracted data set is reduced by neglecting the components associated with small eigenvalues, namely, low frequency components which are related to the non- seizure signal. Given that the volume of the information about the frequency details that is emitted by the epileptogenic area during the seizure is very large, it is possible for the MCSV information to distinguish variations in the frequency information in the non-seizure events as well as onsets of the seizures. Hence, the calculation of the MCSV of each one of the EEG channels reduces a multi-dimensional matrix with a size of 21 × 21 to a vector of size 21 × 1 which is low dimensional. This is accomplished by applying the PCA. This matrix functions as input for the classifier to assist in distinguishing seizure periods from the non-seizure periods of EEG signals.

### 3.6. Classifier

Finally, the robustness of the suggested MCSV method is tested by using classification techniques to produce output labels. This method helps differentiate the undisclosed testing dataset into proper classes depending on the training set details. As a result of subsequent analysis from literature to check the accuracy of the proposed approach, this research paper utilizes three well- known classifiers. Out of the three, two are supervised classifiers, namely, k-NN [30] and SVM [31] and one is an unsupervised classifier namely, K-Means clustering [32].

### 3.7. Data Processing Using IoT- Cloud

**Figure 2:**
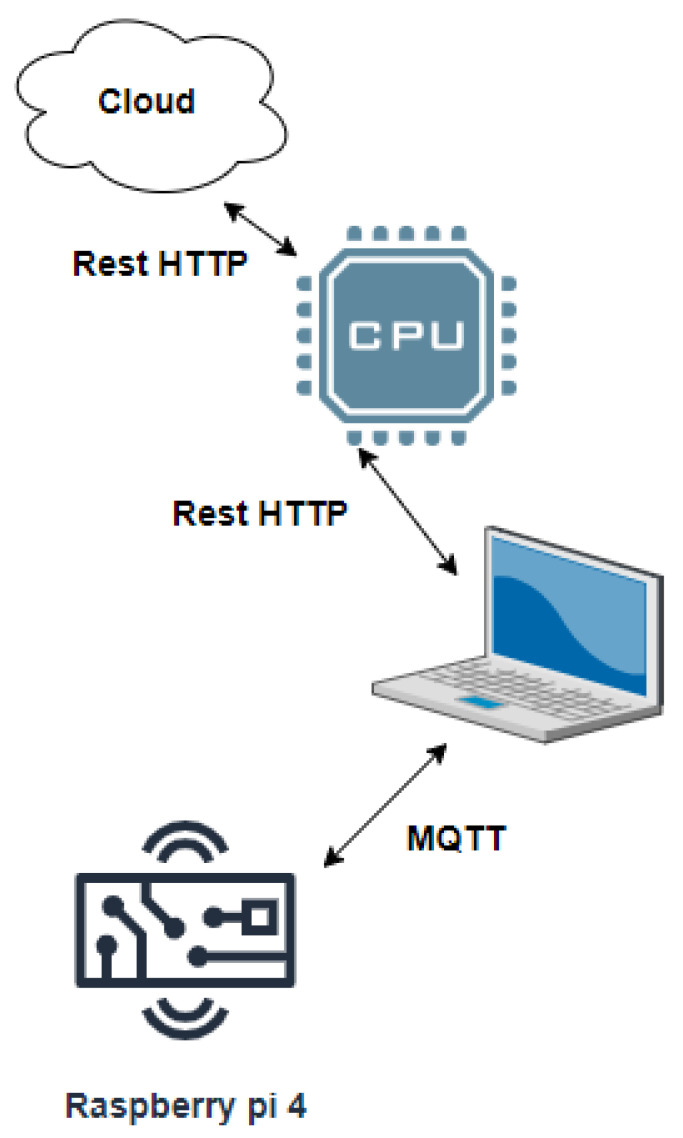
System architecture of the proposed epileptic seizure classification.

In the proposed IoT-cloud based on the MCSV approach, an Android smart phone application (App) was developed which performs the EEG capturing and transmission. Here, the TUH EEG data available in the cloud is directly interfaced with the multichannel EEG acquisition and transmission components. It was followed by the epileptic seizure data processing module which was hosted on the cloud to process the multichannel EEG. This is performed by employing Raspberry-pi 4 and Amazon Elastic Compute Cloud (EC2) using Active Directory (AD) to install the App in the processing unit. From Figure 2, IoT devices are implemented as low-cost processing platforms, Raspberry-pi 4 with Message Queuing Telemetry Transport (MQTT) protocol as a communication protocol, as it is optimized to work on constrained devices. MQTT utilizes a publishers-subscriber model. In order to inform the patient about any abnormal changes ensuing from the EEG signal from the brain lobe, MQTT is made use of. The IoT data generated were transmitted using the Representational State Transfer (REST) HTTP protocol, which provides flexibility and interoperability for developers to create RESTful web services.

## 4. Results

### 4.1. Dataset Description

Epileptic EEG signals are recorded by using scalp electrodes or intracranial electrodes. Various elements impede epilepsy detection. Seizure recordings use scalp electrodes and because of the presence of noises from the scalp, detection of epileptic signals is hampered. However, scalp seizure signals are easier to examine compared to intracranial signals. Hence, the epileptic seizure database used in this paper is obtained using scalp electrodes.

This research work confirms that all experiments were performed following relevant guidelines and regulations. The TUH EEG dataset used for the experimentation is an open access data. Also, this work confirms that the data obtained from the local hospital has been obtained and analysed under the consent and the guidance of Dr. Nandan Yardi, Senior consultant in epileptology and President, Indian Epilepsy Association, Pune chapter, Pune.

#### 4.1.1. Dataset 1

The first dataset used for the analysis of the proposed novel approach comprised records of ten patients with medically complex partial seizures obtained from an open access dataset, Temple University Hospital Electroencephalography Corpus (TUH EEG) [32]. EEG electrodes were placed adapting to the 10–20 electrode placement system. The TUH EEG comprises 21 channels of signals with annotations. The signals were sampled at a frequency range of 250–400 Hz with 16 bits per sample. The file format of the epileptic EEG signal was the European Data Format (EDF+). This version of the database included mainly two types of annotations such as: (i) seizure events including the start-end time, channel label, and seizure category, and (ii) regular or unusual classification of a signal. The label files had event related markings which indicated the information of the occurrence of the event in a specific channel or a set of channels.

#### 4.1.2. Dataset 2

In this study, the second dataset used was obtained from a local hospital in Pune, India, with the consent of the ethical committee. EEG recordings were Unipolar 21 channel recordings obtained from three different patients. The length of the EEG recordings was from one to eight hours. The international 10–20 electrode placement was utilized for placing electrodes and the data were recorded at a sampling rate of 250 Hz. A neuro-expert annotated the data as seizure and non-seizure data. A detailed explanation of the datasets used for the experimentation is displayed in Table 1.

### 4.2. Evaluation Parameters

Initially, the length of the analysis window is the basis for the segmentation of the EEG signals. For the purpose of this study, the authors chose 4 s as the length of the analysis window. A comparison of the outcome of seizure detection acquired by the algorithm proposed in this paper and the results decided by experienced epileptologists was conducted by making use of parameters such as average detection rate, specificity, sensitivity and accuracy [33]. The sensitivity (Sen) was utilized for measuring the capability of detecting ictal periods of EEG segments. The specificity (Spe) was used for measuring the capability of detecting interictal periods, the average detection rate (ADR) was indicated by the means of sensitivity and specificity periods of the EEG segments.

The evaluation parameters are given by (14), (15), (16), and (17), respectively,

(14) $$Sensitivity\left(Sen\right)=\frac{True\;Positive\;\left(TP\right)}{True\;Positive\;\left(TP\right)+False\;Negative\left(FN\right)}$$

(15) $$Specificity\left(Spe\right)=\frac{True\;Negative\;\left(TN\right)}{True\;Negative\;\left(TN\right)+False\;Positive\left(FP\right)}$$

(16) $$Accuracy\;\left(Acc\right)=\frac{TP+TN}{TP+FN+TN+FP}$$

(17) $$Average\;detection\;rate\;\left(ADR\right)=\frac{Sen+Spe}{2}$$

where *TP* denotes the number of seizure periods that are correct; *FP* represents the number of incorrect seizure periods; *TN* denotes the number of correct non-seizure periods; and *FN* shows the number of incorrect non-seizure periods.

The proposed approach was validated making use of a ten-fold cross validation technique [17]. To start with, the EEG signal was partitioned into ten equal portions. Nine out of ten parts were utilized for training and one remaining part was used for testing the performance of the proposed MCSV approach. By moving the training and the testing datasets the strategy used for the validation was iterated ten times. Finally, the sensitivity, specificity, and accuracy were computed as an average of these evaluations.

### 4.3. Selection of Segment Size

Choosing the appropriate length of the segment window as well as the window overlap for the segmentation of the EEG signals was critical. In order to investigate the effect of different lengths of non-overlapping windows on the result of seizure detection, three different window sizes of 2 s, 4 s and 5 s, were considered for analysis in this work.

From this analysis it was observed that there was no significant effect when different segment sizes are used, with or without overlapping of segments in the epileptic identification results. However, the EEG segment should not be too small such that the analysis takes more time. A data length of 4 s with a non-overlapping window was considered as an optimal window size in this proposed work.

### 4.4. Seizure Detection Using the Proposed MCSV Approach

The presence of a seizure event can be seen in the low as well as high frequency bands. It is important to retrieve the seizure information from these frequency bands by filtering out abrupt changes. This purpose was served by the MRAF technique. In addition, the technique was able to localize the epileptogenic area indicating the start and end of seizures present in the seizure signal accurately [29]. Initially the seizure data were segregated into either low or high frequency coefficients, adopting DWT so as to be able to obtain the seizure signal that was present in the frequency band that is the highest. The mother wavelet used is db5 with a four-level decomposition [34]. Then, soft thresholding was executed to achieve the smoothened details of the EEG. Soft thresholding was applied to suppress the abrupt variations existing in the seizure signal. Multiresolution smoothening was achieved as a result of the threshold value computed for various ranges of frequencies [35]. The MRAF method outplayed the soft thresholding performed with DWT by automatically adapting to the high frequency characteristics of an epileptogenesis area. In the annotation shown in Table 1, for example, the seizure of subject 1 starts at 5.15 s and ends at 37.2075 s. Hence, the start of the seizure was at 5.15× sampling frequency, which is a 2060 sample, and terminated at 37.2075× sampling frequency, which is a 14,883 sample. This information regarding the presence of a seizure was properly preserved by the MRAF method. The proposed MCSV approach was tested for three types of classifiers, namely, K-Means clustering, k-NN, and SVM.

**Figure 3:**
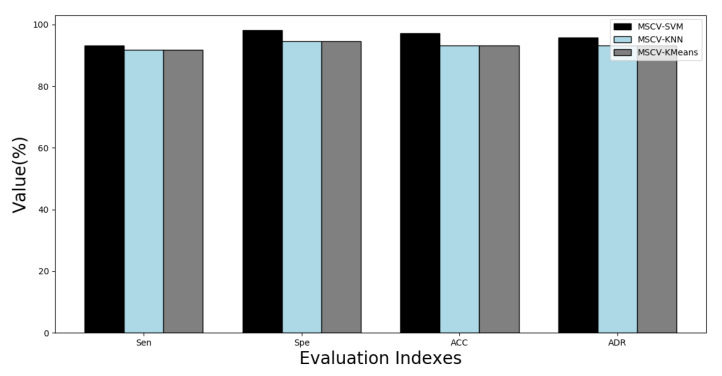
Comparison of four assessment indices for thirteen patients using the MCSV method employing K-Means, k-NN, and SVM as classifiers.

Figure 3 shows comparative results of the K-Means clustering, k-NN, and SVM classifiers for the proposed MCSV approach. The accuracy (ACC) based on the proposed algorithm ranged between 92.99% and 99.45%, and for most of the patients the ACC reached above 98%. The performance of the proposed approach using SVM in terms of the parameter ACC was noted as an average accuracy of 97.38%, the average sensitivity for the method was 93.83%, the average specificity was 97.94% and the average rate of detection was 95.89%. It was observed from the analysis that the average specificity was 62.85%, 88.97%, and 97.94% for K-Means, k-NN and SVM methods, respectively.

From this analysis it was observed that the proposed MCSV approach using the SVM classifier provided a better detection rate as compared to MCSV with K-Means and k-NN as classifiers because it was able to handle outliers. Moreover, the SVM classifier is superior to K-Means and k-NN in two ways: (i) the boundary decision that SVM determined was more accurate; and (ii) the non-linear classification accuracy for SVM was higher. Therefore, we used the MCSV-SVM classifier combination in this work to detect epileptic seizures.

### 4.5. Localization of the Brain Focal Region of the Seizure Activity

**Figure 4:**
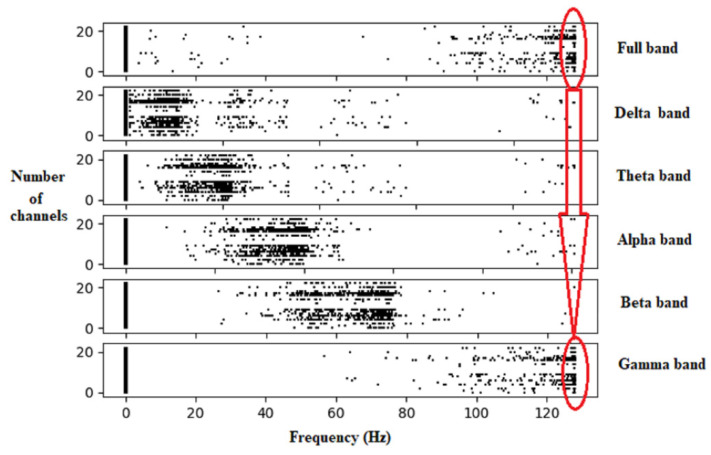
Extraction of high frequency by employing MCSV approach.

The proposed MCSV approach extracted segment-wise multichannel seizure information at each EEG frequency band. Figure 4 shows the result of the frequency information present in the various EEG bands. It can be seen that the MCSV method can pick-up HFOs, above 120 Hz present in the gamma frequency band. As per the work conducted by [24], HFOs act as significant EEG characteristics which confirm the epileptic seizure activity. Also, by using the CSV as the feature, the HFOs are properly chosen by all 21 channels. The presence of HFOs is reflected in the full frequency band information, which contains all frequencies contained in the EEG signal. The other EEG frequency bands are able to identify information related to low frequency content present in the signal. This analysis was further extended to observe the effectiveness of the proposed MCSV approach in the localization of the seizure activity in the brain region, by using a topographic map representation.

**Figure 5:**
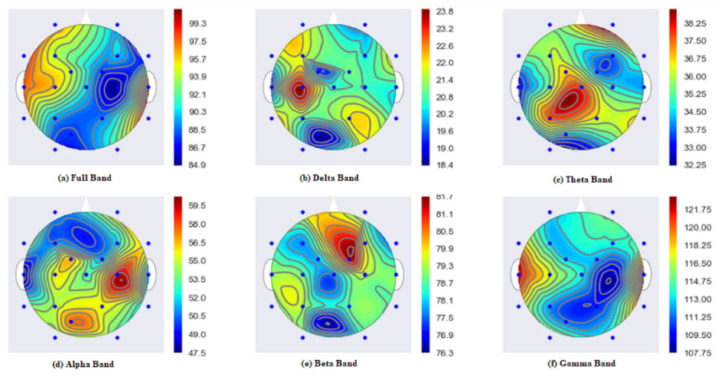
MCSV extracted frequency representation for the estimation of seizure behavior in the scalp, where (**a**) represents the full band; (**b**) gives the delta band activity; (**c**) is the theta band information; (**d**) represents the alpha band signals; (**e**) is the activity in the beta band; and (**f**) represents the activity in the frequency gamma band.

Figure 5 shows the topographic map highlighting the localization of the seizure activity in the brain. Here, the topographic map is plotted using the ggplot Python library. The warmer the color, the stronger is the activity in a particular area. In Figure 5 gamma band and full bands show stronger activity (in red color) originating from the left and right ear lobes. This shows that the power is distributed in the frequency range above 120 Hz, which are HFO activities. This result is again verified with the annotations provided for subject 1. From the dataset annotations for subject 1, for example, the seizure activity starts from the left and right ear lobes. By utilizing the MCSV approach, it is observed from Figure 5 that strong seizure signals are emerging from the ear lobes and are captured by gamma and full frequency bands of the MCSV. Details corresponding to the delta frequency represent the effect of the signal from the ear lobe electrodes. This demonstrates the significance of MCSV approach in the localization of the origin of the seizure region in the brain.

**Figure 6:**
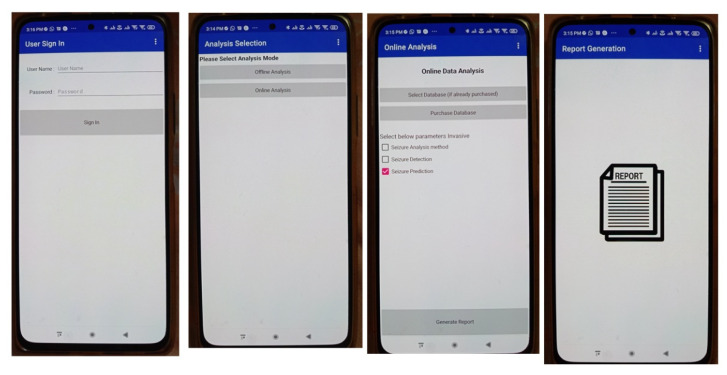
Smart phone application interface with raspberry-pi 4 and Amazon EC2.

Figure 6 shows the data capturing and processing unit, data analysis unit and the report generation part in the App. Here, the data can be captured either in the online or offline mode.

## 5. Discussion

The cloud application was used for reading and storing the EEG sensor data that were input. The IoT system enabled by Raspberry pi 4 was used to store and process the EEG data to enable the user to view it at any time and at any place utilizing a Web browser and an Internet connection. Specific modules like pre-processing and data management, feature extraction and dimensionality reduction, and multichannel classification were processed by the Raspberry pi 4 processing unit. In the proposed work, HTTP was utilized by making use of a GET and POST request to transmit information from the Raspberry pi enabled IoT to the cloud application. In the proposed work, cloud service Storage as a Service (STaaS) was made use of as a host for the cloud application that has the function of storing the EEG signal information and visualizing it.

Segmenting the signal into small sections helps to identify the seizure region accurately in the EEG signal. Segment-wise variation in the spectral power helps to track normal EEG segments from abnormal seizure segments. This research work utilized spectral changes to trace this abnormal seizure information using the MCSV approach. The proposed MCSV approach extracted the actual seizure information using the optimized CSV feature by employing a BI algorithm, namely, FPA. FPA, in comparison to other BI algorithms, has shown better results in terms of flexibility, scalability, and adaptability, especially for non-stationary signals such as an EEG signal. The proposed CSV feature was able to capture HFOs represented by the gamma band of the EEG signal. Detection of origination of HFOs from the brain region facilitated localization of the seizure activity. The proposed MCSV approach was able to capture HFOs thereby precisely localizing the seizure information in the brain region.

The primary goal of this work was to examine HFO characteristics and the utility of the CSV features that are extricated from the EEG signal for classifying seizures early. The MCSV feature employed in this work was made up of six characteristics, namely, full band, gamma, beta, alpha, theta, and delta bands. The percentage of the explained variances for the main components was calculated by taking in to account the abovementioned six bands and then applying the PCA. Results of the investigation indicated that a variance of 81.71% was noted in the first component, 80.93% was noted in the second component, in the third component 43.06% was observed, for the fourth 24.31% was noted, 21.69% for the fifth was observed and for the last a variance of 4.37% was noted. This analysis of variance was undertaken to note how much information is lost with the application of dimensional reduction. The six characteristics were projected in two dimensions. Once the reduction was accomplished, it was noted that there was no specific meaning for each of the components. It was also noted that when fitting the original data to two dimensions, the reduction from six to two dimensions did not permit separation of the classes from each other in an ideal way. Taking into account these observations, it was found that it is not suitable to undertake the reduction for more than two dimensions for classification of the seizure classes.

## 6. Comparative Performance Analysis of the Proposed MCSV Approach

### 6.1. Single Channel vs. Multichannel

To assess the seizure detection functioning of the proposed MCSV approach, multichannel classification was performed against single channel classification using the SVM classifier. The results of MCSV-SVM method for single and multichannel EEG data are summarized in Table 2.

Based on Table 2, the accuracy for the single channel dataset was 96.53%, the sensitivity was 92.8%, the specificity was 98.13%, and the ADR was 95.46%. Whereas the accuracy for the multichannel dataset was 97.38%, the sensitivity was 93.83%, the specificity was 97.94%, and the ADR was 95.89%. As information carried by all channels was considered for classification improvement in terms of sensitivity, specificity, accuracy, and ADR in the EEG seizure analysis, it was observed that more channels from the EEG data carried more information and therefore there was an increase in the sensitivity, specificity, accuracy, and ADR in EEG seizure analysis.

### 6.2. Comparison of the Proposed MCSV Approach with Existing Approach

Various methods for epileptic seizure detection are introduced in literature. A brief exploration of a few of the techniques and their connected investigations with the suggested MCSV-based approach is presented in this section. Table 3 displays this comparative exploration. It can be seen that a majority of the studies highlighted the importance of power spectra-based features and the use of wavelet domain-based information. All these parameters act as significant factors for effective and efficient seizure diagnosis. The work recommended by this paper detected almost all the seizures for all the subjects considered. The MCSV approach helped to attain better seizure detection accuracy in comparison to all the proposed techniques. This may be due to the following reasons: (i) the use of EEG seizure information from multi-channels; and (ii) the proposed MCSV approach is able to capture HFOs representing the seizure information precisely.

In addition, refs. [44,45,46] have attained epileptic seizure detection accuracy of 100%, but they have been unable to locate the exact seizure onset region present in the brain. Also, none of these methods discussed the classification of multichannel EEG signals segment-wise, which play a crucial role in the recognition of seizure activities from the lobes of the brain.

However, it is a challenge to compare the proposed approach with the present techniques, since all the discussed methods have their own method of channel-segment selection, their own type and number of features selected, and type of classifier used for analysis. Nevertheless, it was observed from the literature summary that the MCSV approach reported maximum possible average accuracy by considering classification of the seizure signals for all the EEG channels segment-wise. Even though a few methods report greater accuracy than the presented one, it is worthwhile to note that they have reported the results only for a single channel. In addition to this, few approaches have shown superiority for multichannels by considering only a few channels from the entire EEG channel set. Also, the MCSV approach used all the channels without adapting to any channel selection algorithm. In addition, the MCSV approach enhanced the classifier performance by extracting the high frequency seizure component, which a majority of the existing methods fail to do.

The authors of [33] showed that there is a very close association between high frequency information and epilepsy. It is evident that the MCSV approach can better describe the epileptic behavior present in the seizure signal. As indicated by Figure 5, the maximum seizure spread is indicated clearly in the MCSV obtained gamma and full bands. This highlights the significance of MCSV approach in the proper detection of epileptic seizure.

## 7. Conclusions

A novel MCSV approach for multichannel epileptic seizure classification was presented in this paper. The proposed approach will help in the real-time monitoring of the EEG signal by utilizing an IoT-based system utilizing a smart phone and the cloud. In the data processing unit, the input EEG signal is pre-processed using the MRAF method for the elimination of physiological artifacts. Choice of the segment size greatly affects the performance of the segment-wise seizure classification. Segment-wise analysis was carried out for EEG segments with and without overlap. From this analysis, we observed that a 4 s segment without overlap performs better for seizure classification. The classification performance of the proposed MCSV approach was tested for three standard classifiers, namely, K-Means clustering, k-NN and SVM. It was observed that the classification performance of the MCSV approach with SVM was superior to the K-Means clustering algorithm and the k-NN classifiers. The MCSV-SVM classifier achieved 97.38% accuracy and a 95.89% average detection rate. This result was further analyzed by employing a topographic map in which the gamma band showed stronger activity related to the origination of the seizure from the left and right ear lobes. We also tested the MCSV approach for single and multichannel EEG signals. It was noted from the analysis that multichannel seizure classification carried more information in comparison to a single channel thereby increasing the average detection rate of the epileptic seizure classification. Also, the proposed MCSV approach outperforms other multichannel-based seizure techniques presented in the literature.

## Figures and Tables

**Table 1:** Segment wise Dataset Description.

Subject Index	Dataset	Total No. of Samples	Sampling Frequency (Hz)	No. of Seizures	Start of Seizure (s)	End of Seizure (s)
1	TUH	79,200	400	2	5.15	37.207
2	TUH	135,600	400	2	10.27	142.98
3	TUH	357,000	250	5	83.94	1271.23
4	TUH	400,250	250	10	20.63	1521.94
5	TUH	109,000	250	4	162.1	580.90
6	TUH	518,500	250	3	63.85	1355
7	TUH	373,500	250	17	57.86	1493
8	TUH	302,750	250	7	1	1112.276
9	TUH	96,250	250	4	52.54	374.48
10	TUH	269,250	250	16	1	678.93
11	Local	14,547,500	250	2	12.63	62.14
12	Local	10,412,470	250	4	46.8	192.12
13	Local	192,860	250	1	71.25	118.33

**Table 2:** Classification result based on MCSV-SVM using single and multichannel.

Channel	MCSV-SVM Method			
	Average Values			
	SEN (%)	SPE (%)	ACC (%)	ADR (%)
Single	92.8	98.13	96.53	95.46
Multi	93.83	97.94	97.38	95.89

**Table 3:** A Summary of Literature Presenting Various Techniques for Epileptic Seizure Detection.

Authors with Reference Number	Channel/Segment (in Seconds, s)	Technique Used	Localization of Seizure Origination	SEN (%)	SPE (%)	ACC (%)	ADR (%)
Sriram et al. [36]	Multi/NS	Multifeatures and multi layer perceptron	NO	97.1	97.8	NS	NS
Anubha Gupta et al. [37]	Single/NS	Hurst component and autoregressive moving average based features are used	NO	NS	NS	97	NS
M.G.Moha mmadi et al. [26]	Multi/NS	CNN-MLP/LSTM/ ResNet	NO	76.84/ 94.24	NS	NS	NS
Acharya, U. R. et al. [38]	NS	13 layer CNN	NO	95	90	88.67	NS
Gao et al. [39]	Multi/NS	Maximal overlap DWT	NO	NS	NS	94.12	NS
M.G.Moha mmadi et al. [40]	Multi/0.9 s	HMM-Deep learning	NO	Above 90	95	NS	NS
Zimeng et al. [41]	Multi/NS	Multi-step spike detection algorithm	YES	97.4	96.5	96.9	NS
Cura et al. [42]	Multi/NS	DMD-spectral moments	NO	92.5	98.6	96.5	NS
Yao et al. [43]	Multi/NS	BiLSTM with CNN	NO	87.3	88.3	NS	NS
S.Roy et al. [27]	Multi/NS	Deep Learning	NO	75 to 91.6	NS	NS	NS
The proposed work	Multi/4 s	CSV based MCSV-SVM approach	YES	93.83	97.94	97.38	95.89

NS represents Not Specified.

## Data Availability

The authors are not authorized to share the data publicly.
